# The Current Knowledge of Cerebral Magnetic Resonance Imaging in Monochorionic Twins: A Systematic Review of the Last 20 Years

**DOI:** 10.3390/jcm12237211

**Published:** 2023-11-21

**Authors:** Mathies Rondagh, Enrico Lopriore, Linda S. de Vries, Femke Slaghekke, Lisanne S. A. Tollenaar, Jeanine M. M. van Klink, Sophie G. Groene, Sylke J. Steggerda

**Affiliations:** 1 Department of Neonatology, Leiden University Medical Centre, J6-S, P.O. Box 9600, 2300 RC Leiden, The Netherlands; 2 Willem-Alexander Children’s Hospital, Department of Paediatrics, Division of Neonatology, Leiden University Medical Center, 2333 ZA Leiden, The Netherlands; e.lopriore@lumc.nl (E.L.); l.s.de_vries@lumc.nl (L.S.d.V.); j.m.m.van_klink@lumc.nl (J.M.M.v.K.); s.g.groene@lumc.nl (S.G.G.); s.j.steggerda@lumc.nl (S.J.S.); 3 Fetal Therapy, Department of Obstetrics, Leiden University Medical Center, 2333 ZA Leiden, The Netherlands; f.slaghekke@lumc.nl (F.S.); l.s.a.tollenaar@lumc.nl (L.S.A.T.)

**Keywords:** cerebral injury, magnetic resonance imaging, monochorionic twins, selective fetal growth restriction, single intrauterine fetal demise, twin anemia polycythemia sequence, twin–twin transfusion syndrome

## Abstract

The distinct placental angioarchitecture in monochorionic (MC) pregnancies increases the risk of complications such as twin–twin transfusion syndrome (TTTS), twin anemia polycythemia sequence (TAPS), and selective fetal growth restriction (sFGR). The aim of this systematic review was to evaluate the incidence, type, and severity of cerebral injury and structural brain development on fetal and/or neonatal cerebral magnetic resonance imaging (MRI) in MC twins with or without complications. Twenty-three studies were included, covering a wide range of complications observed during MC pregnancies, with studies involving sIUFD (*n* = 12), TTTS (*n* = 7), mixed complications (*n* = 2), TAPS (*n* = 1), and uncomplicated MC pregnancy (*n* = 1). TAPS and sFGR were largely underrepresented in the current literature. The included studies reported that MC pregnancies with single intrauterine fetal demise (sIUFD) are most at risk for cerebral injury during the fetal period. The overall median incidence of cerebral injury after sIUFD was 28.3% (0–55%). Severe antenatal cerebral injury after sIUFD was detected antenatally in 6.5% (0–36%) of the cases. Three of the included studies described the incidence, type, and severity of cerebral injury on neonatal MRI in MC twins. Structural brain development based on cerebral biometry was only assessed in two studies, revealing significantly smaller biometric measurements of the cerebrum in cases of single sIUFD or smaller twins compared to singleton pregnancies. To enhance our understanding of the potential risks and pathophysiological mechanisms associated with cerebral injury and structural brain development in MC twins, there is a need for future studies and standardized protocols using serial fetal and neonatal MRI imaging in addition to routine ultrasound imaging.

## 1. Introduction

Monochorionic (MC) twin pregnancies are associated with increased perinatal morbidity and mortality compared to singleton or dichorionic pregnancies [1]. The unique placental angioarchitecture of monochorionicity predisposes to important complications, including twin–twin transfusion syndrome (TTTS), twin anemia polycythemia sequence (TAPS), and selective fetal growth restriction (sFGR) [2]. TTTS occurs in approximately 10–15% of MC twin pregnancies and is characterized by an unequal exchange of blood between the twins via placental vascular anastomoses. This leads to polyuria and polyhydramnios in the recipient twin and oliguria and oligohydramnios in the donor twin. Furthermore, the presence of imbalanced blood flow initiates a cascade of progressive hemodynamic disturbances, characterized primarily by cardiac overload in the recipient twin and persistent hypoperfusion and hypoxemia in the donor twin [3]. High blood pressure is a common occurrence in recipients, often observed alongside cardiac overload. Multiple factors are responsible for this phenomenon, such as dysregulation in the renin–angiotensin system and changes in endothelin production [4]. When TTTS is left untreated, mortality rates are as high as 80–90% [5]. Fetoscopic laser surgery of placental vascular anastomoses is a minimally invasive surgical procedure and the most effective first-line therapy for TTTS [6]. TAPS is a rare condition (3–5%) in which chronic transfusion through minuscule vascular anastomoses leads to anemia in the donor and polycythemia in the recipient. This can occur spontaneously or after treatment for TTTS when residual anastomoses are present [1]. Lastly, sFGR is a prevalent (10–15%) complication in which one twin is growth-restricted as a result of an unequally shared placenta, leading to a significant intertwin growth discordance. All these complications can lead to cerebral injury and have proven negative effects on neurodevelopmental outcomes later in life. In some cases, TTTS, TAPS, and sFGR can result in single or double fetal demise. Single IUFD (sIUFD) can have significant consequences for the surviving co-twin, with a high risk of cerebral injury and subsequent neurodevelopmental disabilities due to acute exsanguination in the presence of large patent anastomoses.

Both fetal and neonatal magnetic resonance imaging (MRI) can be used to detect cerebral injury, allowing for early diagnosis, counseling parents, and planning further management [7]. Fetal MRI provides detailed information about early brain development, maturation, and the presence of antenatal brain injury [8,9]. Neonatal MRI can show already established fetal brain injury but also injury due to perinatal and postnatal complications. There is limited information available in the current literature regarding the use of MRI concerning cerebral injury, structural brain development, and neurodevelopmental outcomes in complicated MC twin pregnancies [8].

Therefore, this systematic review aims to evaluate (1) the incidence, type, and severity of the cerebral injury and structural brain development on fetal and/or neonatal cerebral MRI in MC twins and (2) the differences in cerebral MRI findings between pregnancies complicated by TTTS, TAPS, sFGR, and sIUFD.

## 2. Materials and Methods

### 2.1. Search Strategy

This systematic review was conducted using Preferred Reporting Items for Systematic Reviews and Meta-Analyses (PRISMA) guidelines. This systematic review was not registered at PROSPERO. The online electronic Cochrane Library, Embase, PubMed database, and Web of Science were searched in March 2023 for relevant studies published from 1 January 2000 to 7 March 2023. The search strategy consisted of the keywords ‘Monochorionic Twins’ and ‘Magnetic Resonance Imaging’. Additionally, a variety of synonyms were added as free-text words and MESH terms (Appendix A). An information specialist was consulted for the development of the search terms. Reference lists of reviewed articles were manually searched to identify relevant articles that were missed.

### 2.2. Study Selection

All relevant articles were assessed for eligibility through screening of the title and abstract after the removal of duplicates. Thereafter, full-text articles were assessed. All clinical trials, cohort studies, and case–control studies were eligible for inclusion when the cohort consisted of MC twin pregnancies, in which an MRI of the cerebrum was performed in the fetal and/or neonatal period. Articles were excluded when they did not distinguish between MC twins and other exposed groups (e.g., dichorionic twins or singletons), between types of neuroimaging (ultrasound or MRI), or when MRI findings were reported mainly for other organs and only partially for the brain. In addition, articles were excluded when they concerned case reports, small case series (*n* < 3), or conference abstracts; when they were not written in English; or when the full text was unavailable. The search results were assessed independently by two reviewers (M.R. and S.G.). In the case of disagreement, another author (S.S.) was introduced to reach a consensus. The primary outcome was the incidence (both % and exact ratio) of cerebral injury in any degree on fetal and/or neonatal MRI. The secondary outcomes were the incidence of severe cerebral injury, description of the MRI abnormalities, structural brain development based on cerebral biometry, timing of the MRI, and the MRI sequences used. Severe cerebral injury was defined as the presence of intraventricular hemorrhage (IVH) > grade II, periventricular leukomalacia ≥ grade II, a porencephalic cyst and/or periventricular hemorrhagic infarction, arterial ischemic infarction, parenchymal bleeding, ventricular dilation ≥ 97th percentile, basal ganglia, thalamic and/or cortical injury, cerebellar injury, gyration/migration disorders of migrational abnormalities (such as polymicrogyria), extensive encephalomalacia, or other severe cerebral injury associated with an adverse neurological outcome [10].

### 2.3. Quality Assessment

The Newcastle–Ottawa scale for non-randomized studies was used for the quality assessment [11]. This quality assessment is based on three main items: selection, comparability, and outcome. Depending on the score, the quality of the article can be categorized as good, fair, and poor quality (Appendix B). Methodological quality was independently assessed by two authors (M.R. and S.G.), and discrepancies in assessment were resolved through joint discussion until a consensus was reached.

## 3. Results

### 3.1. Study Selection, Quality Assessment, and Study Characteristics

#### 3.1.1. Study Selection

**Figure 1:**
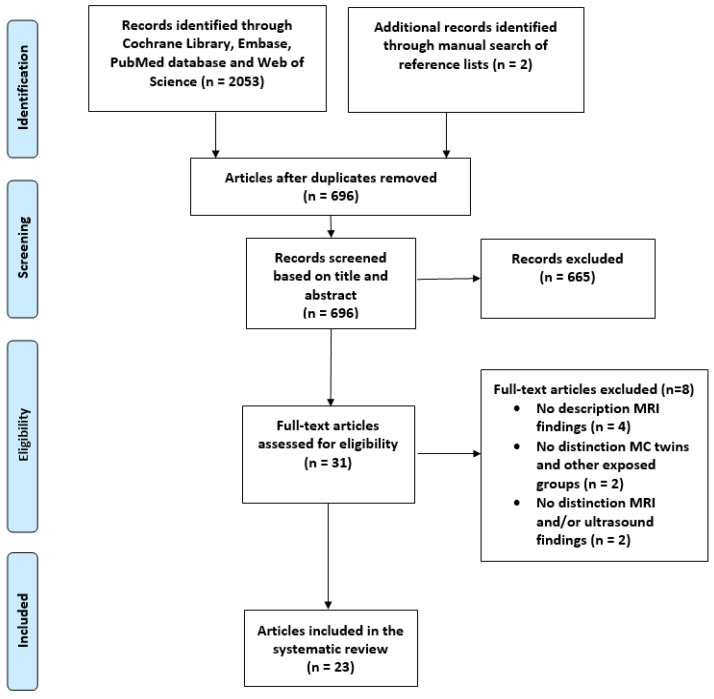
Flow diagram of literature search.

A total of 2053 articles were identified with our search strategy (Appendix A), and a manual search of the reference lists provided two additional articles. After excluding 1357 duplicates, 696 articles were screened based on title and abstract. This primary assessment led to the exclusion of 665 articles. Of the remaining 31 articles, 8 articles were excluded after full-text assessment. The final analysis included 23 articles (Figure 1).

#### 3.1.2. Quality Assessment

The quality of the included studies was evaluated and categorized using the Newcastle–Ottawa scale (Appendix C). Out of the twenty-three studies, twenty (87%) were rated as “good quality”, three (13%) as “fair quality”, and none as “poor quality”. The minimum and maximum scores attained by the studies were 5 and 9 points, respectively. The lowest score was attributed to the absence of clearly defined selection criteria, inadequate comparability, and a lack of detailed follow-up criteria. None of the studies were excluded based on the quality assessment.

#### 3.1.3. Study Characteristics

Twenty-three studies were comprehensively analyzed, covering a wide range of complications observed during MC pregnancies, with studies involving sIUFD (*n* = 12), TTTS (*n* = 7), mixed complications (*n* = 2), TAPS (*n* = 1), and uncomplicated MC pregnancy (*n* = 1).

**Figure 2:**
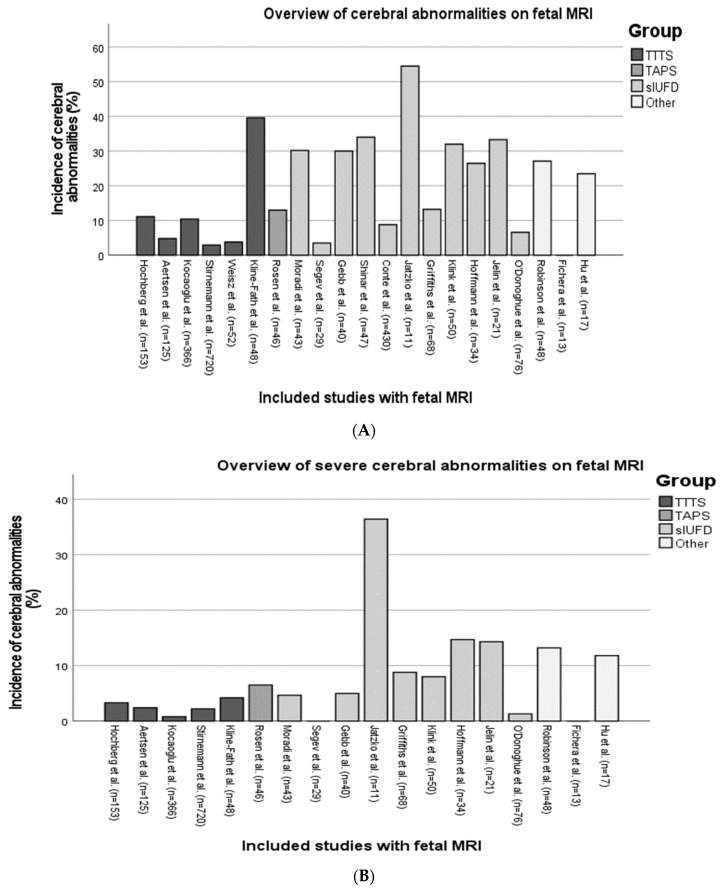
Overview of the incidence of cerebral injury (**A**) and severe cerebral injury (**B**) on fetal MRI in MC pregnancies [7,8,10,12,13,14,15,16,17,20,21,22,23,24,25,26,27,28,29,30,31].

The majority of studies analyzed in this systematic review were published within the past 5 years (*n* = 13), and the first study dated back to 2006 [12]. Out of the included studies, five were prospective cohort studies, and eighteen were retrospective cohort studies (Table 1). A total of twenty-two studies presented fetal MRI findings (Table 2, Figure 2A), and three studies described neonatal MRI findings (Table 3). O’Donoghue et al. and Klink et al. were the only studies that described both fetal and neonatal MRI imaging [10,13]. In nineteen studies, it was possible to indicate/analyze the severity of cerebral injury (Table 4, Figure 2B). Combining the results of all studies, a total of 2733 fetuses or neonates could be assessed for cerebral injury based on their MRI.

**Table 1:** Baseline characteristics of included studies.

First Author (Year)	Country	Study Design	Study Period	Number of Fetuses/Neonates with MRI (*N*)	Population	Validity
Shinar (2022) [14]	International	R	2008–2020	47 ^a^	sIUFD *	Good
Rosen (2022) [15]	Israel	R	2013–2021	46 ^a^	TAPS	Good
Gebb (2022) [16]	USA	R	2009–2021	40 ^a^	sIUFD	Good
Segev (2022) [8]	Israel	R	2017–2020	29 ^a^	sIUFD	Good
Moradi (2022) [17]	Iran	P	NR	43 ^a^	sIUFD	Good
Anh (2022) [18]	Vietnam	P	2019–2021	21 ^b^	TTTS	Good
Halevy (2021) [19]	Israel	P	2010–2015	10 ^a^	Uncomplicated	Good
Hochberg (2021) [20]	Israel	R	2011–2019	153 ^a^	TTTS	Good
Aertsen (2021) [21]	Belgium	R	2010–2017	125 ^a^	TTTS	Good
Kocaoglu (2020) [7]	USA	R	2014–2018	366 ^a^	TTTS	Good
Stirnemann (2018) [22]	France	R	2003–2015	720 ^a^	TTTS	Good
Conte (2018) [23]	Italy	R	2002–2015	430 ^a^	sIUFD	Good
Robinson (2017) [24]	Australia	R	2007–2016	48 ^a^	Mixed (**)	Fair
van Klink (2015) [10]	The Netherlands	R	2002–2013	50 ^a,b^	sIUFD	Good
Jatzko (2015) [25]	Austria	R	2005–2012	11 ^a^	sIUFD	Good
Griffiths (2015) [26]	UK	R	2004–2013	68 ^a^	sIUFD	Fair
Weisz (2014) [27]	Israel	P	2009–2012	52 ^a^	TTTS	Good
Hoffmann (2013) [28]	Israel	P	2007–2010	34 ^a^	sIUFD	Good
O’Donoghue (2009) [13]	UK	R	2000–2007	76 ^a,b^	sIUFD	Good
Fichera (2009) [29]	Italy	R	2001–2006	13 ^a^	sIUFD *	Good
Jelin (2008) [30]	USA	R	1997–2007	21 ^a^	sIUFD	Good
Kline-Fath (2007) [31]	USA	R	2003–2005	48 ^a^	TTTS	Good
Hu (2006) [12]	USA	R	NR	17 ^a^	Mixed (**)	Fair

Abbreviations: NR, not reported; P, prospective cohort study; R, retrospective cohort study; sIUFD, single intrauterine fetal demise; TAPS, twin anemia polycythemia sequence; TTTS, twin-to-twin transfusion syndrome; UK, United Kingdom; USA, United States of America. * MC twins partially consisting of sFGR twins. ** Including TTTS, TAPS, sFGR, co-twin demise after fetoscopic laser surgery, and sIUFD with an unknown cause. ^a^ Fetal MRI. ^b^ Neonatal MRI.

**Table 2:** Characteristics of fetal MRI studies.

First Author (Year)	GA Age at MRI (Weeks)	Population	Incidence of Cerebral Injury in Fetus (%, *n*/*N*)	Cerebral Injury (Number of Observations)	MRI Sequence
Shinar (2022) [14]	25.9 ± 4.2 weeks	sIUFD	34.0 (16/47)	Ischemic injury (16)	T1- and T2-weighted MRI, SWI, and DWI
Rosen (2022) [15]	28–32 weeks	TAPS	13.0 (6/46)	Enlarged dural venous sinuses (1), subependymal blood lateral ventricles (1), severe cerebral ischemia (2), cerebellar hemorrhage (1), asymmetrical lateral ventricles (1)	T1- and T2-weighted MRI and DWI
Gebb (2022) [16]	NR	sIUFD	30.0 (12/40)	Recipients: choroid plexus hemorrhage (2), GM-IVH grade I-II (6), cerebral malformation with PMG and schizencephaly (1); donors GM-IVH grade I (2), PMG (1)	T2-weighted MRI and DWI
Segev (2022) [8]	30.9 ± 1.2 weeks	sIUFD ^ø^	3.5 (1/29)	Cerebral biometry Bilateral caudothalamic cystic changes (1)	T1- and T2-weighted MRI and DWI
Moradi (2022) [17]	21.24 ± 2.29 weeks	sIUFD ^ø^	30.2 (13/43)	GM-IVH (10), extensive cerebral ischemia (2), mild ventriculomegaly (1)	T1- and T2-weighted MRI and DWI
Halevy (2021) [19]	31.3 weeks (IQR 30–33)	Uncomplicated	N/A	Cerebral biometry	T2-weighted MRI
Hochberg (2021) [20]	28–32 weeks	TTTS	11.1 (17/153)	Ischemic brain injury (4), PVH (1), cerebellar hypoplasia (3), sinovenous thrombosis (1), porencephalic cyst (1), GM-IVH (5), ventriculomegaly (3)	T1- and T2-weighted MRI and DWI
Aertsen (2021) [21]	28–32 weeks	TTTS	4.8 (6/125)	Hemorrhagic injury (1), PMG (3), cortical atrophy (2)	T2-weighted MRI and DWI
Kocaoglu (2020) [7]	NR	TTTS	10.4 (38/366)	Diffusion restriction (33), GM-IVH grade I-II (2), grade III-IV (3)	T1- and T2-weighted MRI and DWI
Stirnemann (2018) [22]	30–32 weeks	TTTS	2.9 (21/720)	Bilateral (2) or focal (1) leukomalacia (1), bilateral (1) or focal PMG, severe ischemic injury (8), unilateral schizencephaly (1), severe VM (4), VM with abnormal gyration (1), brain atrophy (1), unknown (1)	T1- and T2-weighted MRI
Conte (2018) [23]	24 weeks (IQR 21–26 weeks)	sIUFD	9.7 (42/430)	PVL (2), generalized (9), posterior (7) or bilateral para-sagittal and peri-Sylvian injury (3), encephalomalacia, focal non-hemorrhagic (14) and hemorrhagic (7) injury	T1- and T2-weighted MRI
Robinson (2017) [24]	25 weeks (IQR 21–29 weeks)	Mixed	27.1 (13/48)	IVH, delayed sulcation, BPD < 5th percentile and bilateral VM (1), bilateral occipital cortical infarction with PMG (1), decreased hemispheric size, asymmetrical ventricles < 10 mm (1), dural sinus thrombosis (1), cystic lesions (1), mildly delayed sulcation (2), encephalomalacia (2), severe abnormal sulcation (1), increased subarachnoid space (2), cerebral biometry <10th percentile (1)	T1- and T2-weighted MRI and DWI
van Klink (2015) [10]	26.5 weeks (IQR 22.3–30.8)	sIUFD	16/50 (32%)	NR	T1- and T2-weighted MRI
Jatzko (2015) [25]	23.5 ± 2.3 weeks	sIUFD	54.5 (6/11)	IVH grade I (2), IVH grade III (2), schizencephaly and several small parenchymal hemorrhages (1), cysts lateral to ganglionic eminence, mild VM (1)	T1- and T2-weighted MRI and DWI
Griffiths (2015) [26]	NR	sIUFD	13.2 (9/68)	Demise with TTTS: mild VM (1), focal infarction with PMG (2), extensive encephalomalacia (1); demise with an unknown cause: mild VM (2), extensive encephalomalacia (3)	T1- and T2-weighted MRI and DWI
Weisz (2014) [27]	NR	TTTS	17.3 ^‡^ (9/52) 3.8 ^‡‡^ (2/52)	Group after fetoscopic laser surgery: GMH (6), ischemia (3); follow-up at 30–32 weeks in same group: cerebral atrophy (1) and cerebral edema compatible with old infarct and ventricular dilation (1)	T1- and T2-weighted MRI and DWI
Hoffmann (2013) [28]	NR	sIUFD ^ø^	26.5 (9/34)	sIUFD with an unknown cause: ischemic injury (2), cerebral edema (1); sIUFD and TTTS: infarction (1), GM-IVH (1), bilateral PVH (1); selective reduction: bilateral cerebral ischemia (1), GM-IVH (2)	T1- and T2-weighted MRI and DWI
Fichera (2009) [29]	20.6 weeks (IQR 19.1–31.5)	sIUFD	0 (0/13)	No cerebral injury	T1- and T2-weighted MRI and DWI
O’Donoghue (2009) [13]	NR	sIUFD ^ø^	6.6 (5/76)	Selective reduction group: focal hemorrhage (1) and moderate VM with aqueduct stenosis (1); group with sIUFD with an unknown cause: MCA infarct (1), GMH (1), mild VM (1)	T1- and T2-weighted MRI
Jelin (2008) [30]	24 weeks and 2 days	sIUFD	33.3 (7/21)	sIUFD with TTTS: unilateral infarct with developing PMG (1), focal injury in left parietal lobe (1), severe destruction of supratentorial brain (1), choroid plexus and posterior fossa subarachnoid hemorrhage (1); TRAP group: bilateral germinolytic cysts, (1) Sylvian fissures slightly shallow (1); demise with an unknown cause: bilateral mild VM; delayed sulcation (1)	T1- and T2-weighted MRI
Kline-Fath (2007) [31]	NR	TTTS	39.6 (19/48)	TTTS donor group: cerebral malformation (2) and cerebral sinovenous enlargement (12); TTTS recipient group: IVH grade I or II/ischemia (2) and cerebral sinus enlargement (3)	T1- and T2-weighted MRI
Hu (2006) [12]	NR	Mixed	23.5 (4/17)	TTTS group: IVH grade IV (1); demise with an unknown cause: IVH grade I (2), porencephalic findings (1)	T1- and T2-weighted MRI

Abbreviations: BPD, biparietal diameter; DWI, diffusion-weighted imaging; GM-IVH, germinal matrix–intraventricular hemorrhage, GA, gestational age; MCA, middle cerebral artery; NR, not reported; PMG, polymicrogyria; PVH, periventricular hemorrhage; PVL, periventricular leukomalacia; sIUFD, single intrauterine fetal demise; SWI, susceptibility-weighted imaging; TAPS, twin anemia polycythemia sequence; TRAP, twin reversed arterial perfusion; TTTS, twin-to-twin transfusion syndrome; VM, ventriculomegaly. ^‡^ Group after fetoscopic laser surgery; ^‡‡^ Group at follow-up at 32 weeks. ø Including single survivors after selective fetal reduction.

**Table 3:** Study characteristics of neonatal MRI studies.

First Author (Year)	Age at MRI (Weeks)	Population	Incidence of Cerebral Injury in MC Twins (%, *n*/*N*)	Cerebral Injury (Number of Observations)	MRI Sequence
Anh (2022) [18]	At birth and 3 and 6 months after birth	TTTS	0 (0/21)	Normal MRI	NR
van Klink (2015) [10]	NR	sIUFD	23.4 (11/47)	Ischemic injury (3), IVH grade II (1), IVH grade III-IV (3), PVL grade III (3), severe cerebral atrophy (1)	T1- and T2-weighted MRI and DWI
O’Donoghue (2009) [13]	NR	sIUFD ^ø^	7.7 (8/104)	Selective reduction: focal hemorrhage (1), moderate VM with aqueduct stenosis; demise with an unknown cause: MCA infarct (1), focal hemorrhage (1), mild VM (1), bilateral ischemic injury basal ganglia (1), widespread cystic lesions (1), injury of the periventricular white matter and basal ganglia and infarct in Sylvian fissure (1)	T1- and T2-weighted MRI

Abbreviations: DWI, diffusion-weighted imaging; MCA, middle cerebral artery; NR, not reported; VM, ventriculomegaly. ø Including single survivors after selective fetal reduction.

**Table 4:** Incidence of severe cerebral injury in fetal or neonatal MRI studies.

First Author (Year)	Population	Incidence of Severe Cerebral Injury (%, *n*/*N*)	Severe Cerebral Injury (Number of Observations)	Neurological Outcome
Shinar (2022) [14]	sIUFD	N/A	NR	NR
Rosen (2022) [15]	TAPS	6.5 (3/46)	Focal restricted diffusion in right thalamus and chronic ischemic changes in frontoparietal areas (1, A). Chronic ischemic changes in frontoparietal areas accompanied by frontal lobe atrophy (1, B), cerebellar hemorrhage (1, C)	A and B: TOP
Gebb (2022) [16]	sIUFD	5.0 (2/40)	Diffuse PMG with primitive sulcation and decreased parenchyma posteriorly (1, A), cerebral malformation with PMG, and schizencephaly (1, B)	A: normal development at 4 months B: global developmental delay, seizures C: epilepsy, developmental delay, and right hemiparesis
Segev (2022) [8]	sIUFD	0.0 (0/29)	0	N/A
Moradi (2022) [17]	sIUFD	4.65 (2/43)	Extensive cerebral ischemia (2)	NR
Anh (2022) [18]	TTTS	0 (0/21)	0	N/A
Halevy (2021) [19]	Uncomplicated	N/A	NR	NR
Hochberg (2021) [20]	TTTS	2.0 (3/153)	Porencephalic cyst (1), IVH grade III (1), unilateral ventriculomegaly with deviation of midline (1)	NR
Aertsen (2021) [21]	TTTS	2.4 (3/125)	Focal PMG (3)	Cerebral palsy at 5 years (1), TOP (1), and unknown (1)
Kocaoglu (2020) [7]	TTTS	0.8 (3/366)	GMH grade III (1) and GMH grade IV (2)	NR
Stirnemann (2018) [22]	TTTS	2.2 (16/720)	Bilateral leukomalacia (2), bilateral polymicrogyria (1), severe ischemic injury (8), Unilateral schizencephaly (1), severe ventriculomegaly (4)	NR
Conte (2018) [23]	sIUFD	N/A	NR	NR
Robinson (2017) [24]	Mixed	13.2 (5/38)	IVH, delayed sulcation, BPD< 5th percentile and bilateral VM (1, A), bilateral occipital cortical infarction with polymicrogyria, cystic lesions (1, B), encephalomalacia (2, C), severe abnormal sulcation (1, D)	A: TOP B: developmental delay C: TOP (1) and palliative care (1) D: developmental delay at 6 months and died at 12 months
Van Klink (2015) [10]	sIUFD	4/50 (8%) * 8/28 (28.6%) **	Fetal MRI: MCA infarction (1, A), bilateral MCA infarction (1, B), multicystic encephalopathy (1, C), severe cerebral atrophy (1, D) Neonatal MRI: cPVL grade III (1), unilateral IVH grade II with infarction caudate nucleus (1), multicystic encephalopathy (2), diffuse cortical necrosis (3), unknown (1)	Fetal MRI group: TOP (2) and survival (2) Neonatal MRI group: neonatal death (4) and survival (4)
Jatzko (2015) [25]	sIUFD	36.4 (4/11)	IVH grade III (2, A), closed schizencephaly and several small parenchymal hemorrhages (1, B), cysts lateral to ganglionic eminence and mild VM (1, C)	A: TOP (1) and neonatal death 2 days after birth (1) B: cerebral palsy at 5 years C: normal clinical assessment at 1 year
Griffiths (2015) [26]	sIUFD	8.8 (6/68)	Focal infarction with PMG (2, A), micrencephaly with extensive encephalomalacia (3, B), and extensive encephalomalacia (1, C)	A: live birth at 38 weeks (1) and stillbirth at 32 weeks (1) B: neonatal death (1), TOP (1), and live birth at 38 weeks (1) C: extensive encephalomalacia: stillbirth at 28 weeks
Weisz (2014) [27]	TTTS	N/A	NR	NR
Hoffmann (2013) [28]	sIUFD	14.7 (5/34)	Severe ischemic injury (2, A), several temporal lobe and periventricular infarcts (1, B), bilateral periventricular hemorrhage (1, C), bilateral cerebral ischemia (1, D)	A: TOP (1) and motor deficiencies at 1 year (1) B, C, and D: TOP
Fichera (2009) [29]	sIUFD	0.0 (0/13)	0	N/A
O’Donoghue (2009) [13]	sIUFD	1.3 (1/76) * 5.3 (4/76) **	Fetal MRI: MCA infarction (1, A) Neonatal MRI: MCA infarction (1, B), bilateral ischemic injury in basal ganglia (1, C), widespread cystic lesions (1,D), injury of the periventricular white matter and basal ganglia and infarct in Sylvian fissure (1, E)	A: mild hemiplegia B: mild hemiplegia C: mild bilateral dystonic hemiplegia D: severe neurodevelopmental abnormality E: normal at 1 year
Jelin (2008) [30]	sIUFD	14.3 (3/21)	Unilateral infarct with developing PMG (1), severe destruction of supratentorial brain (1), hemorrhage bilateral choroid plexus and posterior fossa and subarachnoid hemorrhage adjacent to cerebellum (1)	NR
Kline-Fath (2007) [31]	TTTS	4.2 (2/48)	Cerebral malformation (2)	NR
Hu (2006) [12]	Mixed	11.8 (2/17)	IVH grade IV (1), porencephalic findings (1)	NR

Abbreviations: BPD, biparietal diameter; GMH, germinal matrix hemorrhage; MCA, middle cerebral artery; NR, not reported; PCM, polymicrogyria; PVL, periventricular leukomalacia; TOP, termination of pregnancy; VM, ventriculomegaly. * Fetal MRI group. ** Neonatal MRI group.

The results are methodically structured as follows: firstly, a concise overview of fetal MRI findings is provided for each distinct group; following this, neonatal findings are elaborated upon. Subsequent to this, a comprehensive critical analysis pertaining to structural brain development was conducted, and finally, a summation of the timing, sequences used, and field strength of the MRI was undertaken.

### 3.2. Fetal MRI Findings in MC Twins

#### 3.2.1. Twin–Twin Transfusion Syndrome

Six out of twenty-three studies described fetal MRI findings in MC twin pregnancies complicated by TTTS [7,20,21,27,31,32]. In terms of quantity, this resulted in the largest group studied, comprising a total of 1937 fetuses that were scanned using fetal MRI. Overall, the median reported incidence of cerebral injury in TTTS was 7.6% (range 2.9–39.6%). Of the six studies, one study performed fetal MRI prior to fetoscopic laser surgery, four studies performed MRI scans post-fetoscopic laser surgery, and the timing of the MRI in the remaining study was not reported. None of the studies performed amnio drainage as a treatment for TTTS. Four studies investigated the correlation between Quintero stages and the risk of cerebral injury; however, no significant correlation was found [7,20,22,27]. Kocaoglu et al. reported abnormal MRI findings in 10.4% (38/366) of fetuses before fetoscopic laser surgery in TTTS [7]. Cerebral injury was primarily seen in donor twins (*n* = 25) and consisted of germinal matrix hemorrhage and cerebral infarction. Hochberg et al. reported that 11.1% (17/153) of fetuses (nine donors and eight recipients) had cerebral injury after fetoscopic laser surgery, and this rate was higher when laser surgery was performed later during gestation (between 20 and 23 weeks as compared to 16–19 weeks) [20]. This study reported on both severe cerebral injuries, such as IVH grade 3 (*n* = 1), porencephalic cysts (*n* = 1), and severe ventriculomegaly (*n* = 1), but also on other injuries, such as mild ischemic brain injury (*n* = 4), sinovenous thrombosis (*n* = 1), GM-IVH (*n* = 4), ventriculomegaly (*n* = 2), and cerebellar hypoplasia (*n* = 3). Aertsen et al. observed cerebral injury in 4.8% (6/152) of MC twins after fetoscopic laser surgery, including five donor twins and one recipient [21]. This consisted of migrational abnormalities (e.g., polymicrogyria) and atrophy due to previous acute or subacute ischemia. Stirnemann et al. reported a 2.9% (21/720) incidence of cerebral injury following fetoscopic laser surgery, diagnosed in thirteen donor twins and nine recipients, consisting of brain atrophy, IVH, severe ventriculomegaly, and migrational abnormalities [22]. Weisz et al. identified cerebral injury in 17.3% (9/52) of MC twins 1–5 days after laser surgery [27]. This study demonstrated two types of injury: small hemorrhages (e.g., transient, minor bleeding in the germinal matrix and basal ganglia) and acute brain ischemia and infarction. The number of donor and recipient twins with cerebral injury was not described. Kline-Fath et al. reported that 39.6% (19/48) of MC twins (24 donors and 24 recipients) had abnormal cerebral MRI findings associated with TTTS, with more injury in donor twins compared to recipients [31]. However, it was not clear whether this was before or after intervention. The abnormal findings consisted mostly of cerebral venous sinus enlargement without thrombosis (*n* = 12) but also mild IVH and ischemia. Two neonates had severe cerebral malformations (2/48; 4.2%).

#### 3.2.2. Twin Anemia Polycythemia Sequence

Rosen et al. was the only study that retrospectively examined cerebral injury on MRI in fetuses affected by TAPS (*n* = 46) [15]. Twenty-six (56.5%) cases had spontaneous TAPS, and in twenty cases (43.5%), TAPS developed after laser treatment for TTTS. This study did not provide details regarding the distribution of cerebral injury between donor and recipient twins. Pregnancies were followed with fetal brain ultrasound, and fetal MRI was performed when there was a suspicion of cerebral injury between 28 and 32 weeks of gestation. Neonatal brain imaging included cranial ultrasound. This study revealed cerebral injury in 13.0% (6/46), which constituted four donors and two recipients. The observed cerebral injury included intracerebral hemorrhage (*n* = 2), ischemic injury (*n* = 2), dural venous sinus thrombosis (*n* = 1), and asymmetrical lateral ventricles (*n* = 1). The authors concluded that TAPS may place fetuses and neonates at increased risk for cerebral injury compared with uncomplicated monochorionic twins.

#### 3.2.3. Selective Fetal Growth Restriction

None of the studies included in this review focused solely on the incidence of cerebral injury on fetal MRI in sFGR twins unless the pregnancy was complicated by sIUFD (see Section 3.2.5). Moradi et al. investigated the role of MRI, including DWI, in the detection of acute ischemic cerebral injury in MC pregnancies complicated by TTTS and treated with fetoscopic laser surgery [17]. This study also included 11 fetuses (25.6%) with isolated sFGR without TTTS. Altogether, 30.2% (13/43) of fetuses exhibited cerebral injury on MRI. Because the authors did not differentiate between the subgroups, a statement about the impact of sFGR alone, without sIUFD, on cerebral injury was not possible.

#### 3.2.4. Single Intrauterine Fetal Demise

Twelve out of twenty-three studies described fetal MRI findings in surviving MC co-twins after sIUFD [8,13,14,16,23,25,26,28,29,30]. This resulted in the second largest group studied with fetal MRI, with brain imaging findings reported in 862 fetuses. Of note, in 111 cases, sIUFD was caused by selective fetal reduction. The studies reported a median overall incidence of cerebral injury in single survivors of 28.3% (range 0–55%) after sIUFD [8,13,14,16,17,23,25,26,28,29,30]. We made a distinction between studies with single survivors after selective fetal reduction and other causes of sIUFD because of the different mechanisms of cerebral injury.

Eight studies in surviving co-twins after sIUFD excluded cases with selective fetal reduction [10,14,16,23,25,26,29,30]. These studies reported a median incidence of cerebral injury in single survivors, including survivors after selective fetal reduction, of 31.0% (0–55%) after sIUFD.

Shinar et al. reported that 34.0% (16/47) of the cases had evidence of cerebral injury, of which TTTS survivors exhibited the highest incidence of ischemic injury (6/13; 46.2%), followed by uncomplicated MC twins (5/13; 38.5%), and sFGR twins (5/21; 23.8%) [14]. Gebb et al. described that 30% (12/40) of the survivors in pregnancies complicated by sIUFD after fetoscopic laser surgery had abnormal MRI findings, with the highest risk after the demise of the recipient (50%) rather than the donor twin (14%) [16]. Of these 12 survivors with abnormal MRI findings, 9 were classified with mild injury (e.g., germinal matrix hemorrhage or IVH grade I) and 3 with severe injury (e.g., IVH grade II, diffuse PMG, and cerebral malformation). In another study, Conte et al. reported MRI abnormalities in 9.7% (42/430) of the surviving co-twins [23]. The most common abnormalities were non-hemorrhagic focal injury and generalized encephalomalacia. However, detailed information about the severity of cerebral injury was not reported. Van Klink et al. described that 32.1% (16/50) of the surviving MC co-twins had cerebral injury [10]. In most of these cases, pregnancy was complicated by TTTS (35%), sFGR (27%), or monoamniocity (10%). In 8% (4/50) of the fetuses, MRI showed severe cerebral injury, including middle cerebral artery infarction (*n* = 2), multicystic encephalomalacia (*n* = 1), and severe, generalized cerebral atrophy (*n* = 1). Jatzko et al. described an incidence of cerebral injury in 55% (6/11) of survivors of MC twin pregnancies after sIUFD, including IVH grade 1 (*n* = 2), IVH grade 3 (*n* = 2), schizencephaly (*n* = 1), and cysts lateral to ganglionic eminence with mild ventriculomegaly (*n* = 1) [25]. Griffiths et al. reported an incidence of cerebral injury of 13.2% (9/68) in cases with TTTS or an unknown cause of fetal demise [26]. The rate of cerebral injury was similar in both groups, respectively, 4/27 (14.8%) and 5/41 (12.2%). The most common cerebral abnormality was ventriculomegaly with/without encephalomalacia and less frequently PMG or micrencephaly [26]. Fichera et al. reported no signs of cerebral injury in 13 surviving co-twins after sIUFD [29]. Finally, Jelin et al. reported that 33.3% (7/21) of the survivors exhibited cerebral injury after sIUFD associated with TTTS, twin reversed arterial perfusion (TRAP), or unknown diagnosis [30]. Various cerebral abnormalities were detected, including polymicrogyria, germinolytic cysts, intracranial hemorrhage, ventriculomegaly, and delayed sulcation.

The following four studies also included single survivors after selective fetal reduction [8,13,17,28]. These studies reported a median incidence of cerebral injury in single survivors, including survivors after selective fetal reduction, of 16.5% (3.4–30.2%) after sIUFD. Segev et al. described only one case (3.4%) with cerebral abnormalities among 29 MC pregnancies with sIUFD, including 14 fetuses with TTTS and sIUFD after fetoscopic laser surgery [8]. The only fetus with cerebral abnormalities had bilateral caudothalamic cystic changes. However, their cohort also included eight cases with selective reduction. Hoffmann et al. reported cerebral injury in 26.5% of cases (9/34) with sIUFD. This study divided the cases into three groups. Cerebral injury was observed in fetal MRI with an unknown cause of the sIUFD (3/6), in sIUFD associated with TTTS (3/10), and sIUFD associated with severe complications and selective reduction (3/18) [28]. Altogether, five fetuses exhibited severe cerebral ischemia and infarction, while the other four cases presented with minor findings. O’Donoghue et al. reported the incidence of cerebral injury after sIUFD [13]. The study categorized into three distinct groups: fetuses after sIUFD due to selective reduction (*n* = 45), fetuses after sIUFD due to complicated fetoscopic laser surgery (*n* = 10), and fetuses after sIUFD with an unknown cause (*n* = 21). The overall incidence of cerebral injury was 6.6% (5/76). Survivors of an sIUFD with an unknown cause had the highest risk (3/21) when compared to cases with selective reduction (2/45) and with fetoscopic laser surgery for TTTS (0/10). Cerebral injury in the survivors consisted of focal cerebral hemorrhage (*n* = 1), mild ventriculomegaly (*n* = 2), cerebral artery infarction (*n* = 1), and germinal matrix hemorrhage (*n* = 1). Moradi et al. reported that 30.2% (13/43) of single survivors, after selective reduction by umbilical cord coagulation, had cerebral injury [17]. These twins were diagnosed with TTTS (*n* = 19), sFGR (*n* = 11), fetal reduction (*n* = 2), TRAP (*n* = 1), or major structural anomalies (*n* = 10).

#### 3.2.5. Other Complications

Two studies assessed the role of MRI in identifying cerebral injury in complicated MC pregnancies that did not fit into any of the aforementioned groups [12,24]. In Robinson et al., the cohort consisted of MC pregnancies with sIUFD and complicated pregnancies without sIUFD [24]. Hu et al. did not mention the underlying complication but only stated that the pregnancies were ‘complicated’ [12]. Robinson et al. used MRI to gather additional diagnostic information on co-twin survivors after sIUFD with an unknown cause (*n* = 10), co-twin survivors after sIUFD after treatment with fetoscopic laser surgery (*n* = 8), or twins pairs complicated with TTTS, sFGR, and/or TAPS without sIUFD (*n* = 30) [24]. They reported that altogether 27.1% (13/48) of cases had a cerebral injury. Hu et al. reported on the usefulness of MRI in detecting cerebral injury in various complications of multifetal gestations [12]. They described that 23.5% (4/17) of complicated MC twin pregnancies had cerebral injury detected by MRI.

### 3.3. Neonatal MRI Findings in MC Twins

The number of studies reporting on neonatal MRI findings in MC twins was limited to three studies and 172 neonates. Several studies were excluded from analysis because of (1) a combination of MRI and ultrasound findings, making it unclear which modality specifically detected the abnormality, and (2) the lack of differentiation between MC and dichorionic pregnancies, making it impossible to distinguish between the two groups. Anh et al. and O’Donoghue et al. both performed postnatal MRI scans in complicated MC twins with TTTS and sIUFD, respectively [13,18]. Anh et al. studied 19 cases of TTTS who underwent fetoscopic laser surgery. No cerebral injury was observed [18]. O’Donoghue et al. investigated cerebral injury in survivors of pregnancies following sIUFD and included cases with an unknown cause of the sIUFD (*n* = 27), cases treated with fetoscopic laser surgery (*n* = 14), and after selective reduction (*n* = 63) [13]. This study reported neonatal cerebral injury in 7.7% (8/104) of the survivors. The number of survivors with cerebral injury after procedure-related sIUFD (i.e., laser surgery or selective reduction) was significantly lower (2.6%, 2/77) compared to sIUFD with an unknown cause (22.2%, 6/27). Van Klink et al. evaluated the incidence, type, and severity of neonatal cerebral injury in surviving MC twins after sIUFD [10]. sIUFD was associated with monoamniocity (5/49), sIUGR (13/49), TTTS (17/49), or unknown cause (14/49). Cerebral injury was observed in 23.4% (11/47) of the cases, of which eight (16%) neonates had severe cerebral injury.

### 3.4. Structural Brain Development in MC Twins

Structural brain development on fetal or neonatal MRI was only reported in two studies and based on fetal MRI images [8,19]. In total, 39 fetuses underwent cerebral biometric assessment. Structural brain development in MC twins complicated by TTTS, TAPS, sFGR, or sIUFD in the neonatal period was not reported. Segev et al. reported that supratentorial and infratentorial biometric measures such as fronto-occipital, biparietal, and transcerebellar diameter were significantly smaller in single survivors after sIUFD when compared to singletons. Halevy et al. reported cerebral biometry on fetal MRI in MC twins (*n* = 10) with birth weight discordance (>15% growth discordancy) [19]. The delineation of cerebral structures was performed by a semi-automated algorithm. The volume discordance between the brain structures of the appropriate gestational age twin and the smaller twin was less than the birthweight discordance. Furthermore, the brain volumes of the growth-restricted twins were smaller compared to singletons, while the brain volumes of the larger twins were more likely to be within normal percentiles.

### 3.5. Timing, Sequences Used, and Field Strength of the MRI

Fetal MRI imaging was most frequently performed during the second trimester (*n* = 8), followed by the third trimester (*n* = 6). In seven studies, it was unclear at which gestational age MRI was performed, but the timing was specified (e.g., after intervention or after sIUFD). Three studies performed postnatal MRI. In one study, the timing of MRI was 3–6 months after birth; in the other studies, the timing was not specified. All fetal MR studies included T2-weighted MRI sequences; T1-weighted sequences were included in all except one [19]. In thirteen studies, DWI images were obtained. In fifteen studies, MRI was performed on a 1.5 T scanner; three studies used both 1.5 T and/or 3 T; and one study used only a 3 T MRI scanner. In three studies, the field strength of the MRI scanner was not specified.

## 4. Discussion

### 4.1. Key Findings

This systematic review shows that MC twins are at risk of developing cerebral injury during pregnancy. MC pregnancies complicated by sIUFD (either in combination with TTTS, TAPS, sFGR, or unknown causes) exhibit the highest incidence of cerebral injury on fetal MRI in 28.3% (range 0–55%) of the cases. Moreover, MC pregnancies complicated by TTTS but without sIUFD also carry relatively high incidences of cerebral injury. Severe cerebral injury during the fetal period was identified in 6.5% (0–36%) of cases across different types of complications. It should be noted that cerebral injury in MC twins, with or without complications, is also influenced by various other factors, including the degree of prematurity, birth weight, and postnatal complications. Cases with TAPS and sFGR were largely underrepresented in the current literature. This systematic review shows that there is a significant knowledge gap regarding the incidence, type, and severity of cerebral injury in the neonatal period in complicated MC pregnancies. Moreover, there is a lack of MRI studies regarding neonatal developmental aspects of the brain in MC twins. Comparing the various groups was challenging due to retrospective designs, heterogeneity among studies, varying definitions, limited information, and different indications among studies to conduct an MRI.

### 4.2. Strengths and Limitations of This Study

This systematic review was conducted according to standardized PRISMA methodology and used the validated Newcastle–Ottawa scale for the quality assessment of the studies. It provides a comprehensive overview of the current literature on the use of fetal and neonatal MRI in MC twin pregnancies complicated by TTTS, sFGR, and TAPS and sIUFD. This systematic review included 23 studies and 2733 cases. Due to the structured approach and the large number of cases, it provides valuable information for clinicians in the counseling of parents about the potential risk of cerebral injury during MC twin pregnancies. Our results should be interpreted with caution due to the limitations that were identified in the current literature: (1) largely retrospective study designs which are susceptible to information bias, (2) small and heterogeneous sample sizes, (3) the use of different definitions for cerebral injury across studies, (4) lack of detailed information about the severity of cerebral injury, and (5) different indications for fetal or neonatal MRI resulting in higher incidences of cerebral injury when MRI scans were conducted exclusively following the detection of cerebral injury on ultrasound. Therefore, a comparison between the selected studies was difficult. Another limitation is that only one study prospectively performed serial fetal/neonatal neuroimaging. The underrepresentation of MC pregnancies complicated by TAPS and sFGR is an important limitation. Additionally, there was a lack of studies investigating the structural brain development in MC twins. It is worth noting the possibility of a potential overlap between the multicenter study conducted by Conte et al. and the retrospective single-center study undertaken by Griffiths et al. [28,30].

### 4.3. Interpretation of Findings

#### 4.3.1. Twin–Twin Transfusion Syndrome

The incidence of cerebral injury in fetuses with TTTS ranged from 3 to 40%, with injury being diagnosed both before and after fetoscopic laser surgery. Kocaoglu et al. reported that MC twins with TTTS already had a cerebral injury in 10% prior to fetoscopic laser surgery [7]. Due to the lack of studies that conducted both pre- and post-fetoscopic laser surgery MRI, the exact timing of the injury and the role of the procedure itself remains unknown [20,21,22,27]. Ischemic injury was the most frequent type of injury in cases with TTTS. GM-IVH and ventriculomegaly were also common. The precise pathophysiological mechanisms underlying cerebral injury in MC pregnancies complicated by TTTS are not yet fully understood [21,22]. One hypothesis is that hemodynamic imbalances can cause a wide range of abnormalities, from the early development of polymicrogyria to hypoxic-ischemic injuries [21,22,31]. Additionally, hypoxic-ischemic injuries may be complicated by hemorrhage, which can ultimately result in atrophy, schizencephaly, or porencephaly, depending on the phase of fetal brain development and the timing and severity of TTTS [21]. Donors and recipients appear to be equally at risk for cerebral injury [33]. Previous research reported that in TTTS survivors treated with fetoscopic laser surgery, the incidences of severe neurodevelopmental impairment and cerebral palsy were 3% (7/241) and 2% (4/258), respectively, at two years of age [33]. Also, the incidence of long-term neurodevelopmental impairment in TTTS survivors treated with amnioreduction is high (20%) compared to survivors treated with fetoscopic laser surgery (10%). This significant difference in outcome is partly due to the higher rate of severe prematurity in TTTS treated with amnioreduction. A common hypothesis posits that cerebral injury during fetoscopic laser surgery may arise from compromised cerebral perfusion during the procedure, leading to ischemic or hemorrhagic cerebral injury [22,27]. Another study demonstrated an association between perioperative fetal hemodynamic changes in TTTS pregnancies treated with laser surgery and subsequent poor neurological outcomes [34].

#### 4.3.2. Twin Anemia Polycythemia Sequence

There is limited literature available regarding fetal or neonatal MRI studies in patients with TAPS. Rosen et al. reported that 13% of the fetuses with TAPS, primarily recipients (5/7), already showed cerebral injury before birth [15]. TAPS is a specific type of chronic feto-fetal transfusion that is distinguished by substantial differences in hemoglobin levels between the twins without the presence of the twin oligo-polyhydramnios sequence. The pathophysiology of TAPS involves the existence of a limited number of minuscule arteriovenous anastomoses, enabling gradual blood transfusion from the donor to the recipient and resulting in progressive anemia in the donor and polycythemia in the recipient [3]. The hypothesis regarding hemorrhagic injury in TAPS fetuses proposes that polycythemia contributes to vascular sludging and consumption of coagulation factors, thereby obstructing the sinuses and impeding the in- and outflow of blood from the brain. This obstruction can potentially lead to the development of cerebral sinovenous thrombosis. However, the exact mechanisms underlying cerebral injury remain inadequately understood. Previous research on long-term outcomes in TAPS twins identified bilateral deafness due to auditory neuropathy spectrum disorder in 15% of the donors [35]. The development of auditory neuropathy spectrum disorder involves multiple factors, such as prematurity and perinatal hypoxia; however, the exact pathogenesis is not yet fully understood. Theoretically, the prolonged lack of oxygen during fetal anemia may have caused damage not only to the brain but also to the developing auditory nerve system. Another hypothesis is that certain TAPS donors have reduced albumin levels, which could lead to heightened bilirubin toxicity, potentially causing kernicterus and consequent impaired auditory functions [36]. Neonatal MRI might be useful to determine the underlying pathophysiological mechanisms of this bilateral deafness.

#### 4.3.3. Selective Fetal Growth Restriction

No studies have reported on cerebral injury on fetal or neonatal MRI in MC pregnancies complicated by sFGR. A systematic review focusing on neonatal ultrasound reported an incidence of severe cerebral injury, which varied between 0 and 33% [37]. The highest risk was seen in fetuses with an intermittent absent or reversed end-diastolic flow (A/REDF) in the umbilical artery, otherwise known as Gratacós type III [38]. This particular group may be more susceptible to in utero feto-fetal blood transfusions through large arterio-arterial connections, which can lead to hypoxic injury, especially in the larger twin. Another study indicated that abnormal umbilical artery Doppler measurements significantly increased the incidence of cerebral injury compared to normal umbilical artery Doppler measurements, supporting this hypothesis [37]. An alternative theory is that cerebral injury in the larger twin occurs after birth due to (iatrogenic) premature delivery and subsequent immaturity of the brain, which may lead to prematurity-related complications, including GM-IVH and white matter injury. In MC twins with sFGR, neonatal cerebral ultrasound examinations indicate a consistent and proportional discordance in brain growth [39]. The smaller co-twin exhibits reduced size in cerebral structures, decreased white/deep gray matter, and smaller overall brain-size measurements. Notably, there exists a positive linear correlation between the degree of birth weight discordance and the extent of discordance in intracranial volume. In the long term, the smaller twin frequently demonstrates mild neurodevelopmental impairment and lower developmental test scores. The underlying mechanisms involved in the alterations in brain development observed in the smaller co-twin remain unclear.

#### 4.3.4. Single Intrauterine Fetal Demise

The incidence of cerebral injury in single survivors after sIUFD in MC pregnancies ranged between 0 and 55%. Discrepancies in the incidence of cerebral injury were evident between cohorts, with survivors after selective fetal reduction (16.5%) and without selective fetal reduction (31.0%). Hemorrhagic injury and encephalomalacia were the most frequent types of cerebral injury reported in MC pregnancies after sIUFD. The exact mechanism leading to these different types of cerebral injury is still unclear and strongly depends on the specific cause of sIUFD [10,16]. In the case of selective reduction, it is expected that there will be less brain damage and likely another mechanism for cerebral injury as compared to a group where sIUFD is caused by complications such as TTTS, sFGR, or monoamniocity. Currently, the common hypothesis, except in sIUFD after selective reduction, is that co-twin morbidity results from acute exsanguination from the surviving cotwin into the low-pressure circulation of the dead co-twin [10]. Acute exsanguination results in acute hemodynamic changes, like hypovolemia and anemia, leading to hypoxic-ischemic injury and multiorgan failure [10,16]. Previous studies indicated that a lower gestational age at birth is a recognized risk factor for cerebral injury, specifically IVH, with prematurity being a significant contributor to the incidence of such cases [10]. The size of placental vascular anastomoses tends to increase as gestational age progresses. According to this hypothesis, we speculate that the severity of acute exsanguination could potentially increase with increasing gestational age due to the presence of larger vascular anastomoses, which offer reduced vascular resistance. Interestingly, in MC pregnancies complicated by TAPS, there is no risk of exsanguination after sIUFD due to the presence of small diameter (<1 mm) anastomoses [3]. In the event of acute exsanguination in MC pregnancies with sIUFD, intervention is often too late. A recent study indicated that intrauterine transfusion does not offer better outcomes compared to expectant approaches [40]. Hence, it is vital not to hasten the delivery of the surviving fetus since the damage has already occurred. Instead, a fetal MRI after 2–4 weeks should be performed to determine the management approach.

#### 4.3.5. Structural Brain Development

Conclusions regarding brain development based on fetal or neonatal MRI in MC pregnancies cannot be drawn due to the limited available evidence, consisting of only two small cohort studies involving a total of 39 fetuses [8,19]. To date, no research has investigated structural brain development in MC pregnancies affected by TAPS and sFGR. The studies included in this systematic review stated that several brain structures were significantly smaller in single survivors after sIUFD and smaller twins compared to singletons [8,19]. MC pregnancies affected by sIUFD experience transient hemodynamic fluctuations and reduced brain perfusion, which can potentially impact brain maturation [8]. It is plausible that underlying pathology affects various areas of the developing brain in surviving twins following sIUFD.

### 4.4. Implications for Clinical Practice and Research

To better understand the potential risks and the pathophysiological mechanisms underlying cerebral injury in MC pregnancies, particularly those complicated by TAPS and sFGR, more research is needed. Prospective studies are of utmost importance to investigate the timing, incidence, type, and severity of cerebral injury in complicated MC pregnancies. These studies should include serial fetal and neonatal ultrasound, as well as fetal and neonatal MRI scans. Additionally, to gain insight into the long-term clinical implications of cerebral injury, standardized long-term follow-up assessments of neurodevelopmental outcomes should be included. These results should be compared with control groups of single infants or MC twins without complications with similar characteristics. In the case of TTTS, studies should focus on the relationship between cerebral injury and interventions such as fetoscopic laser surgery. Therefore, it is essential to perform serial fetal ultrasound and MRI both prior to and following fetoscopic laser surgery. In the case of TAPS, studies should focus on the relationship between cerebral injury and the development of bilateral deafness. There is also a need for standardized protocols for the timing and follow-up of fetal and neonatal MRIs. These protocols should include standardized time points of assessment, e.g., a fetal MRI at 30 weeks gestational age and a neonatal MRI at 40–42 weeks postmenstrual age, to allow comparison in the literature. In the case of fetoscopic laser surgery, the implementation of pre- and post-operative MRI scans would provide valuable insights. In addition, there is a need for unambiguous definitions of cerebral injury that describe both the type of injury and the severity. Another noteworthy topic for future research is structural brain development in MC twins. Studies should aim to more precisely elucidate the parameters for typical and atypical brain development during the fetal and neonatal periods. Furthermore, quantitative MRI measurements may serve as a biomarker that could potentially contribute to the prediction of long-term (cognitive) outcomes. International collaboration between fetal therapy centers can play a vital role in advancing our understanding of the mechanisms underlying cerebral injury and their association with neurodevelopmental outcomes. Establishing an international web-based registry for MC pregnancies complicated by TTTS, TAPS, and sFGR would be highly valuable for future research endeavors. This has the potential to assist in better identification of contributing risk factors, genetic susceptibilities, and environmental influences.

## 5. Conclusions

This systematic review shows that MC twins are at increased risk of cerebral injury in the fetal and neonatal period. Yet, there is a lack of studies where MRI is performed with a routine and structured approach, resulting in variability in incidence and patterns of cerebral injury. Most studies focus on TTTS and sIUFD, while there are limited studies reporting on TAPS and sFGR. The knowledge gaps encountered in this review arise from the need to rely on retrospective studies, small and varied sample sizes, inconsistencies in definitions of cerebral injury, and insufficient data on the severity of cerebral injury. Future prospective studies, preferably in multicenter settings with standardized protocols using both serial cerebral ultrasound and MRI imaging during the fetal and neonatal period, should be performed to provide more insight into the potential risk and pathophysiological mechanisms underlying cerebral injury in MC pregnancies. Fetal and neonatal MRIs play an important role in the early detection, assessment, and treatment planning for fetuses or neonates with cerebral injury, which offers valuable benefits to both healthcare professionals and parents.

## Data Availability

All data generated or analyzed during this study are included in this article. Further inquiries can be directed to the corresponding author.

## Appendix A

Search strategy for the different databases.

Cochrane
(“Monozygotic Twin” OR “Monozygotic Twins” OR “Identical Twin” OR “Identical Twins” OR “Monochorionic”):ti,ab,kw AND (“MRI” OR “Magnetic Resonance Imaging” OR “Diffusion-weighted imaging” OR “DWI MRI” OR “fMRI”):ti,ab,kw
07-03-2023: 7 results
Embase
(exp monozygotic twins/OR “Monozygotic Twin”.ti,ab. OR “Monozygotic Twins”.ti,ab. OR “Identical Twin”.ti,ab. OR “Identical Twins”.ti,ab. OR “Monochorionic”.ti,ab.) AND (exp Magnetic Resonance Imaging/OR “Magnetic Resonance Imaging”.ti,ab. OR “MRI”.ti,ab. OR “Diffusion-weighted imaging”.ti,ab. OR “DWI MRI”.ti,ab. OR “fMRI”.ti,ab.) NOT (conference OR conference abstract OR “conference review”).pt. AND 2000:2024.(sa_year).
07-03-2023: 940 results
PubMed
(“Twins, Monozygotic” [Mesh] OR “Monozygotic Twin” [tw] OR “Monozygotic Twins” [tw] OR “Identical Twin” [tw] OR “Identical Twins” [tw] OR “Monochorionic” [tw]) AND (“Magnetic Resonance Imaging” [Mesh] OR “Magnetic Resonance Imaging” [tw] OR “MRI” [tw] OR “Diffusion-weighted imaging” OR “DWI MRI” [tw] OR “fMRI” [tw]) AND (“2000”[Date-Publication]: “3000”[Date-Publication])
07-03-2023: 627 results
Web of Science
TS = (“Monozygotic Twin” OR “Monozygotic Twins” OR “Identical Twin” OR “Identical Twins” OR “Monochorionic”) AND TS = (“Magnetic Resonance Imaging” OR “MRI” OR “Diffusion-weighted imaging” OR “DWI MRI” OR “fMRI”) AND PY = (2000-2024)
07-03-2023: 479 results

## Appendix B

Quality assessment of non-randomized studies included in this systematic review.

**Newcastle**–**Ottawa Scale**
Selection

(1) Representativeness of the exposed cohort
  (a) Truly representative (one star)
  (b) Somewhat representative (one star)
  (c) Selected group
  (d) No description of the derivation of the cohort
(2) Selection of the non-exposed cohort
  (a) Drawn from the same community as the exposed cohort (one star)
  (b) Drawn from a different source
  (c) No description of the derivation of the non-exposed cohort
(3) Ascertainment of exposure
  (a) Secure record (e.g., surgical record) (one star)
  (b) Structured interview (one star)
  (c) Written self-report
  (d) No description
  (e) Other
(4) Demonstration that outcome of interest was not present at the start of the study
  (a) Yes (one star)
  (b) No

Comparability (tick one or both boxes, as appropriate)

(1) Comparability of cohorts on the basis of the design or analysis controlled for confounders
  (a) The study controls for age, sex, and marital status (one star)
  (b) The study controls for other factors (list) ___________ (one star)
  (c) Cohorts are not comparable on the basis of the design or analysis controlled for confounders

Outcome

(1) Assessment of outcome
  (a) Independent blind assessment (one star)
  (b) Record linkage (one star)
  (c) Self-report
  (d) No description
  (e) Other
(2) Was follow-up long enough for outcomes to occur
  (a) Yes (one star)
  (b) No

Indicate the median duration of follow-up and a brief rationale for the assessment above:____________________

(3) Adequacy of follow-up of cohorts
  (a) Complete follow-up—all subjects accounted for (one star)
  (b) Subjects lost to follow-up, unlikely to introduce bias—number lost less than or equal to 20% or description of those lost suggested no different from those followed (one star)
  (c) Follow-up rate of less than 80% and no description of those lost
  (d) No statement

Thresholds for converting the Newcastle–Ottawa scales to AHRQ standards (good, fair, and poor):
Good quality: three or four stars in the selection domain AND one or two stars in the comparability domain AND two or three stars in the outcome/exposure domain.
Fair quality: two stars in the selection domain AND one or two stars in the comparability domain AND two or three stars in the outcome/exposure domain.
Poor quality: 0 or 1 star(s) in the selection domain OR 0 stars in the comparability domain OR 0 or 1 star(s) in the outcome/exposure domain.

## Appendix C

**Table A1:** Results of the quality assessment of the studies using the Newcastle–Ottawa scale.

First Author (Year of Publication)	Selection				Comparability	Outcome			Total
	Representativeness (*)	Non-Exposed Cohort (*)	Exposure (*)	Outcome of Interest (*)	Comparability of Cohorts (**)	Assessment (*)	Follow-Up (*)	Adequacy (*)	
Shinar et al. (2022) [14]	*	*	*	-	**	*	*	-	7/9
Rosen et al. (2022) [15]	*	*	*	*	**	*	*	*	9/9
Gebb et al. (2022) [16]	*	*	*	-	**	*	*	*	8/9
Segev et al. (2022) [6]	*	*	-	*	**	*	*	*	8/9
Moradi et al. (2022) [17]	*	*	-	-	**	*	*	*	7/9
Anh et al. (2022) [18]	*	*	*	*	**	*	*	*	9/9
Halevy et al. (2021) [19]	*	*	*	*	**	*	*	-	8/9
Hochberg et al. (2021) [20]	*	*	*	-	**	*	*	*	8/9
Aertsen et al. (2021) [21]	*	*	*	*	**	*	*	*	9/9
Kocaoglu et al. (2020) [7]	*	*	*	-	**	*	-	*	7/9
Stirnemann et al. (2018) [22]	*	*	-	*	**	*	*	*	8/9
Conte et al. (2018) [23]	*	*	*	-	**	*	*	*	8/9
Robinson et al. (2017) [24]	-	*	*	-	**	*	-	*	6/9
van Klink et al. (2015) [10]	*	*	*	-	**	*	*	*	8/9
Jatzko et al. (2015) [25]	-	*	*	*	**	*	*	-	7/9
Griffiths et al. (2015) [26]	*	-	*	-	*	*	*	*	6/9
Weisz et al. (2014) [27]	*	*	*	-	**	*	*	*	8/9
Hoffmann et al. (2013) [28]	*	*	-	*	*	*	*	*	7/9
O’Donoghue et al. (2009) [13]	*	*	*	-	**	*	*	-	7/9
Fichera et al. (2009) [29]	*	*	*	-	**	*	-	*	7/9
Jelin et al. (2008) [30]	*	*	*	-	*	*	-	*	6/9
Kline-Fath et al. (2007) [31]	*	*	*	-	**	*	-	*	7/9
Hu et al. (2006) [12]	-	*	*	-	*	*	-	*	5/9

Abbreviations: G, good quality; F, fair quality; P, poor quality; *, one star.
